# Rheumatology-led pregnancy clinic: men perspective

**DOI:** 10.1007/s10067-020-05551-0

**Published:** 2021-01-15

**Authors:** Yasser El Miedany, Deborah Palmer

**Affiliations:** 1 King’s College, London, England; 2 Rheumatology Department, North Middlesex University Hospital, London, England

**Keywords:** Autoimmune rheumatic diseases, Biologic therapy, DMARDs, Fertility, Men, Pregnancy

## Abstract

The birth of reproductive rheumatology as a subject of interest in rheumatology has led to improvement of clinical care for patients living with autoimmune rheumatic diseases and paved the way towards setting a specialized pregnancy service within the standard rheumatology practice. In contrast to women, where there has been wealth of literature regarding pregnancy, lactation, and birth outcomes, there is not as much focusing on male sexual health and outcomes among inflammatory arthritis patients. Challenges such as decrease ability to conceive, impaired fertility, erectile dysfunction, and other sexual problems have been raised by male patients living with autoimmune rheumatic diseases. This broad scope gives the reproductive health concept in men another expansion with views to include sexual health problems screening among men attending the standard outpatient rheumatology clinics. This article adds to the paucity of real-life experience and aims at discussing the sexual health from the men perspective and provides a practical approach towards screening, and assessment of men living with autoimmune diseases in standard day to day practice.

## Introduction

Reproductive health and family planning in the context of autoimmune rheumatic diseases (ARDs) can be a challenge for both men and women. In fact, family planning and sexual health are important considerations not only for people living with ARD, but also for the treating health care professionals [1]. Unfortunately, so far these issues are rarely comprehensively addressed in clinical practice and there are unmet needs dealing with such relatively sensitive personal matters in the standard face to face patients’ care [2–6].

From men’s perspective, the impact of ARD on their sexual health and fertility is rather complex. ARD and its medical management may have an impact on several aspects of a man’s reproductive health including the following: (1) sexual function which entails both sexual/physical ability (reflecting body functions), and intimate relationships (reflecting participation and activity) [7]; (2) fertility and gonadal functions; (3) family planning and contraception; and (4) male-mediated teratogenicity and birth outcomes. Earlier data [4, 5, 8] revealed that 36–70% of men living with inflammatory arthritis sustain impaired sexual health attributed to their disease. Furthermore, a tendency to decreased ability to conceive was present in patients living with ARD, which was attributed to sexual problems [9]. Therefore, it is expected that most of these aspects, if not all, are picked up and dealt with by the treating rheumatologist.

The birth of reproductive rheumatology as a subject of interest in the rheumatology field has led to further expansion of clinical care for ARD patients and paved the way towards setting a specialized pregnancy clinic within the standard rheumatology practice. The main aim of these clinics is to deal with the patients’ concerns regarding disease activity and its impact on their personal sexual abilities, fertility, and the perceived negative effect of antirheumatic medications on pregnancy and birth outcomes.

Though there have been some research studies published discussing the ARD impact on different aspects of reproductive health in men, none has considered the implementation of such challenge in standard clinical practice. This article will discuss approaches to handle this challenge starting with the impact of autoimmune rheumatic disease on the man’s sexual dysfunction and ending with a suggested algorithm toward how to assess and manage these patients in the day to day standard rheumatology service.

### Method: search strategy

A narrative review was conducted a series of literature searches in the database MEDLINE/PubMed for English language articles focusing on Pregnancy, contraception, men with rheumatic diseases. The search strategy included a combination of medical subject headings and keywords. The search terms that we used were “Pregnancy” “contraception,” “men,” “autoimmune rheumatic diseases,” “medications,” “erectile dysfunction,” “pregnancy rate,” “pregnancy outcome,” “sperm,” “semen,” “spermatozoa,” “sperm quality,” “rheumatoid arthritis,” “systemic lupus erythematosus” (SLE), “ankylosing spondylitis,” “antiphospholipid syndrome” (APS), and “systemic sclerosis.” Preference was given to the sources published within the past 7 years. Data extraction was carried out by the investigators using a standardized data collection form with subsequent discussion among the authors. Peer-reviewed observational controlled and non-controlled studies (case–control and cohort studies) were selected. All studies were referral center-, hospital-, or population-based studies. The data collected in the selected articles were all related to fertility abnormalities in male patients with ARDs. Articles that were case reports and those that did not evaluate male patients were excluded.

**Fig. 1:**
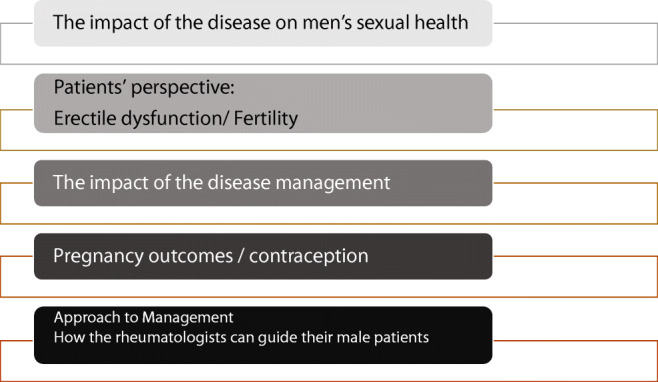
The main steps in handling men’s sexual health in rheumatology-led pregnancy clinic

Figure 1 shows the main steps in handling men’s sexual health in a rheumatology-led pregnancy clinic which will form the main pillars of this review.

### The impact of the disease

There are several aspects involved in male sexual dysfunction which includes having diminished libido and erectile dysfunction (unable to have or maintain sufficient erection to have sexual intercourse over at least a six month period) as well as abnormal ejaculation [10, 11]. In general, chronic ARDs impact negatively on the patient’s personal life with negative effects on the patients’ quality of life, functional ability, ability to have or plan for a family, and causing emotional disturbance. This has been endorsed by earlier data supporting the link between the person’s self-esteem and body image on one side and the patient’s body pains, restricted joint movements, and fatigue on the other side. Sexual problems have been linked also to antirheumatic medications as well as steroids [2].

When it comes to disease activity impact in specific ARDs, previous research has established that sexual disorders may be influenced differently by disease activity parameters, particularly in men. For example, sexual desire and satisfaction are influenced more by pain and depression, while sexual disability is influenced more by physical incapacity [10]. There are no significant differences reported between spondyloarthritis and rheumatoid arthritis regarding their negative impact on man’s sexual health; in fact, both behave in similar manner. For example, in concordance with rheumatoid arthritis, it has been reported that patients with active ankylosing spondylitis, have sexual dysfunction closely associated to their disease activity status, levels of fatigue, functional impairment, quality of life and psychological status particularly depression [11]. Furthermore, similar to DAS-28 in rheumatoid arthritis, BASDAI, but not ESR or CRP, as a measure of disease activity in spondyloarthritis, has been reported to be associated with erectile dysfunction [12]. Similarly, in SLE, an increased prevalence of impotence in men has also been reported [13]. In systemic sclerosis, erectile dysfunction is a common, but often underestimated, clinical feature in men with SSc. The prevalence of ED in SSc has been reported as ranging from 12 to 81% in different studies [11–16].

### Patient’s perspectives

#### Erectile dysfunction

Erectile dysfunction is the most common form of sexual impairment reported in men, and causes them to feel frustrated, shocked, stressed and get overwhelmed with the feeling of emasculation. In standard outpatient rheumatology practice, erectile dysfunction represents a major hurdle to having a healthy marital relationship [2]. In an earlier report [17] a patient described erectile dysfunction as “a shock and a threat to his sense of masculinity.” The research work revealed that while a cohort of the male patients related their sexual dysfunction to having intense pain during sexual activity, others saw it as a side effect of medications they are taking. None of the participants included in that study [17] had been given information regarding the risks of erectile dysfunction as a side effect of prescribed medications or that it may be related to their disease activity. These factors add to the stress and anxiety the patient may feel with a net result of impaired sexual health.

Considering the pathophysiology, erectile dysfunction in ARDs has been linked to endothelial dysfunction. Strong evidence accrued in the last years endorses the concept that erectile function is an exceptional surrogate marker of systemic health in general and vascular performance in particular [18]. This has been described by the equation: endothelial dysfunction = erectile dysfunction (ED = ED, and vice versa) [19]. High levels of pro inflammatory cytokines, specifically TNF-α involved in most of the ARDs pathogenesis, have been suggested to be related to sexual dysfunction and fatigue [20, 21]. In a study of 383 males without rheumatic disease, Matos et al. [22], found that there was a significant link between erectile dysfunction and high levels of TNF- α. It is worth mentioning that the pro-inflammatory cytokines are also closely linked to levels of serum testosterone. Earlier data revealed that hypogonadal males tend to have higher concentration levels of TNF-α, IL-6, and IL-1β as a result of impaired suppression. This ultimately worsens the endothelial dysfunction, with consequent further impairment of erectile function [23]. Therefore, the use of anti-TNF medications could help with improving erectile dysfunction [22, 24].

Another factor that plays an important role in erectile dysfunction among men with ARDs is the presence of associated comorbidities. A close link between cardiovascular risk and erectile dysfunction has been reported [2, 23]. Unfortunately, this is not usually considered in the same context as the more traditional cardiovascular conditions which include hypertension, ischemic heart disease, dyslipidaemia, diabetes mellitus and insulin resistance/metabolic syndrome complex. The good news is that there are now established, regularly updated specific guidelines for the treatment of men with erectile dysfunction who are known to have cardiovascular disease [25].

Lastly, while it is extremely rare that methotrexate therapy induces erectile dysfunction, however, it has been reported that intramuscular methylprednisolone injections given to men have a strong correlation with erectile dysfunction [2].

#### Fertility

The potential of decreased fertility is not unusual concern among ARD patients whether males or females [26, 27]. Several research studies addressed this problem in association with individual rheumatic diseases including rheumatoid arthritis, systemic lupus erythematosus, ankylosing spondylitis, gout, Behçet disease, and dermatomyositis [28–31]. In men living with ARDs, the potential of impaired reproduction has been linked to both the disease directly as well as the medical therapy. The testicular tissue has been identified as the main pathological site [28]. A systematic review was carried out by Tiseo et al [32], to assess the potential alteration of male fertility in different rheumatic diseases. Results revealed that the frequency and severity vary among the different rheumatic diseases. Systemic lupus erythematosus clearly affects gonadal function impairing spermatogenesis mainly due to antisperm antibodies and cyclophosphamide therapy. Behçet disease, gout, and ankylosing spondylitis patients, including those under anti-TNF therapy in the latter disease, do not seem to have reduced fertility whereas in dermatomyositis, the fertility potential is hampered by disease activity and by alkylating agents. Data regarding rheumatoid arthritis revealed that gonadal dysfunction was observed as consequence of disease activity and antisperm antibodies.

Immunologic Infertility, characterized by the presence of antisperm antibody (ASA), has also been reported in men living with ARDs [28]. With the presence of multiple antisperm antibody, this may induce the immobilization and agglutination of spermatozoa, blocking sperm-egg interaction. This can also impede implantation or cause the arrest of the development of the embryo [33, 34]. In rheumatoid arthritis patients with difficulty to conceive, gonadal impaired function with elevated LH/FSH, has been linked to having a higher incidence of ASA [35]. However, although ASA has been found to be present in as many as 42% of male patients with SLE, the real significance of this in infertile men remains controversial and at present there is no standardized treatment regimen [36]. Another point to consider in immunologic infertility, men who present with severally compromised spermatogenesis, kariotype should be evaluated to complete the fertility analysis as aneuploidies are frequent and may also contribute for fertility impairment in SLE patients [37].

On another front, another main factor for gonadal dysfunction is drug treatment. Some drugs, such as nonsteroidal anti-inflammatory drugs in women, and sulfasalazine/methotrexate in men can cause reversible infertility, whereas after treatment with alkylating agents such as cyclophosphamide-CYC and chlorambucil, irreversible infertility has, on occasions, been observed in both genders. Therefore, alkylating agents should be used at the lowest possible dose and alternative therapies, such as azathioprine or mycophenolate mofetil, need to be considered when fertility is an issue [38].

### The impact of the disease management

#### Medications and its compatibility for men with ARDs

There are variations in the concerns regarding the medications used for men living with ARDs. This can be stratified into those men who are planning for a family and those whose sexual partner is pregnant. The concerns prior to conception focus on the effects the medication may have on the man’s fertility as well as the possibility of medication associated teratogenicity. Unfortunately, published information looking at these issues is scarce. Meanwhile, stopping paternal medication needs to be carefully considered, weighing this up with the effect this may have on the disease activity. Concerns after the man’s partner become pregnant includes consideration of whether his medication is present in the seminal fluid, can transfer through the vaginal mucosa to cross the placenta and be teratogenic. As seminal concentrations of medications and volumes transferred are small, hence, post-conception exposure of the embryo or fetus is likely to be minimal [39]. Reassurance can be given when the man’s partner is pregnant, and that there is low risk associated the ARDs treatment. This suggestion has been supported by the findings of the Norwegian birth data and DMARD registry studies. These revealed that there was no risk identified of preterm delivery, low birth weight or congenital abnormalities when evaluating risk of paternal DMARD exposure near conception in comparison to the reference population [40]. This patient cohort included those taking MTX, sulfasalazine, leflunomide, azathioprine, hydroxychloroquine, and TNF inhibitors. A summary of drug compatibility with paternal exposure is shown in Table 1.

**Table 1:** A summary of drug compatibility for men with rheumatic and musculoskeletal disease who are planning to father a child (data based on BSR and ACR guidelines [41, 42])

Compatible with paternal exposure		Not compatible/discontinue	Unable to make a recommendation due to limited data
Recommend continuing	Continue (but limited data)		
Corticosteroids: Prednisolone Methylprednisolone Hydroxychloroquine Azathioprine < 2 mg/kg/day Colchicine Tumor necrosis factor inhibitors (all)	Cyclooxygenase 2 Inhibitors MTX < 20 mg/week Leflunomide Mycophenolate mofetil Mycophenolic acid Cyclosporine Nonsteroidal anti-inflammatory drugs Rituximab Tacrolimus Sulfasalazine (with 5 mg folic acid) (semen analysis if delayed conception/conception may be enhanced by stopping SSZ for 3 months prior to conception)	- Cyclophosphamide (discontinue 12 weeks prior to attempted conception) - Thalidomide (discontinue 4 weeks prior to attempted conception)	Tocilizumab* Abatacept* Apremilast Baricitinib Belimumab* Secukinumab Tofacitinib Ustekinumab

*Unlikely to be harmful [42]

Though the package insert of methotrexate advises the user to wait for at least 3 months (a period which corresponds to the length of the spermatogenic cycle (74 days) [43]), after discontinuing the medication before trying to conceive [36]. However, recent data from a prospective cohort study on paternal MTX exposure, reassuringly, revealed that there was no increase in spontaneous abortions, major birth defects, low birth weight, or low gestational age at delivery [44, 45].

As far as sulfasalazine is concerned, it has been reported to be associated with decreased sperm count, motility, and abnormal sperm morphology [46]. Therefore, male patients could be advised to stop sulfasalazine, particularly if their disease is well controlled or there has been some difficulty with fertility. Temporary stoppage of sulfasalazine therapy in such cases may help to achieve successful conception.

In the case of azathioprine/mercaptopurine paternal exposure before conception, there has been no association with congenital abnormalities [44, 47]. In the study done by Teruel and colleagues [48], assessment of pregnancy outcomes among men with IBD who had taken azathioprine for 3 months prior to conception, did not reveal any significant difference in time to conception, spontaneous abortions or birth weight in comparison to those who did not.

Regarding leflunomide, there is only limited data for paternal exposure to the medication; however, there was an earlier case report of pregnancy outcome of a normal-term pregnancy in the partner of one man who continued to take leflunomide throughout the pregnancy [49].

In SLE and vasculitis, it is more common to use cyclophosphamide to suppress the inflammatory process. Cyclophosphamide has been reported to have negative impact on sperms [50]. Analysis of published data revealed that there was almost universal agreement of reduced sperm counts among men taking cyclophosphamide therapy. The pattern of affection included both azoospermia and teratospermia which was reported whether during or after treatment. On a time-scale, sperm counts tend to drop within the first 3 weeks of therapy, but normally the fall is noted after four months of therapy. Studies which monitored patients after stopping cyclophosphamide therapy reported some improvement in sperm counts over time, although this was not universal [47, 48]. A mean recovery time of 31 months was reported in one study [51] and successful conceptions were reported in five cases [52].

Regarding biologic therapy, the available data regarding the TNF inhibitors and their impact on spermatogenesis is conflicting. One study reported that ten men treated with Infliximab for IBD were found to have an increase in semen volume compared to their figures recorded prior to therapy [50]. However, there was reduction in the percentage of sperm motility as well as the normal oval forms in this cohort of patients [53]. In comparison, there was one case report of oligoasthenozoospermia in a man treated with Adalimumab [54]. When treatment with Adalimumab was stopped sperm morphology and concentration returned to normal; however, the sperm motility remained low. On assessment of men living with spondyloarthritis and receiving anti-TNF therapy, there were no differences in sperm concentration, morphology, or quality reported on comparing patients treated with anti-TNF therapy to healthy controls [55]. This study supports the continued use of anti-TNF therapy in patients who are trying for pregnancy with their partner.

#### Medications and pregnancy outcomes

In general, poor pregnancy outcomes have not been reported among fathers who have been diagnosed to have one of the ARDs [36]. However, considering medical management and its impact on the birth outcomes, there may be some implications to be considered.

##### DMARDs

Several studies assessing pregnancy outcomes where there has been peri- conception paternal exposure to Methotrexate have come to the conclusion that there was no additional risk to the outcome of the pregnancy [56, 57]. The was supported by the outcomes of a large population-based cohort study [58] carried out to look at the longer-term outcomes in 209 children following paternal exposure to Methotrexate. Results did not reveal any negative impact on selected outcomes including malignancies, autism spectrum disorders/schizophrenia/psychosis, and attention deficit hyperactivity disorder. Although a negative impact on spermatogenesis has been reported with the use of sulfasalazine overall pregnancy outcomes following paternal conception seem reassuring [59]. There is no available data regarding paternal exposure to leflunomide and pregnancy outcomes.

##### Anti-TNF therapy

Pregnancy outcomes with paternal TNF exposure have overall been positive. In a study where 47 peri-conception paternal exposures to etanercept were assessed, results did not raise any concern for specific pregnancy outcomes [8, 59]. Similarly, no differences were reported in the percentage of live births or congenital abnormalities among paternal exposures to TNF inhibitors vs other treatments in in the TREAT registry for infliximab for management of Crohn’s disease [60]. Four patients who were treated with Infliximab for ankylosing spondylitis had six healthy children between them. Also, out of ten pregnancies where the male partner was exposed to Infliximab, nine resulted in live births and one miscarriage was reported [61]. In concordance, three cohort studies [61, 62] which included in total 432 men and looked at peri-conceptional paternal exposures to a variety of anti-TNF medications including adalimumab, etanercept, certolizumab, and infliximab did not find any association between these medications and adverse pregnancy outcomes or congenital malformations. The malformation rate in a cohort of 1198 men treated with DMARDs, including 6 men who received adalimumab therapy, was approximately 3.7%, a figure which was quoted to be within average “western countries” rate of 2.4–4% [63].

##### Rituximab

Pregnancy outcomes were recruited from a small number of partners of 16 males receiving rituximab therapy. Follow-up conception period ranged from 2 weeks to 1 year following rituximab infusion. Of 22 pregnancies, outcome data was available in only 11, and included 7 live births (with only 3 gestational ages reported as full term), 2 miscarriages, and 2 ongoing pregnancies [64]. Therefore, in agreement with the BSR guidelines [41], the available evidence, which is based on such limited data, it can be concluded that Rituximab is compatible with paternal exposure

##### Abatacept

In spite of the limited data available, no concerns were reported in relation to peri-conception paternal exposures to abatacept therapy. Assessment of pregnancy outcomes in a cohort study of 10 peri-conception paternal exposures to abatacept [65], results revealed 9 live births and 1 elective abortion, with no congenital abnormalities or fetal deaths.

#### Teratogenicity

Limited data in men suggests that although male factor teratogenicity can occur by direct drug effect on sperm development or with seminal fluid exposure during intercourse, paternal use of antirheumatic drugs other than cyclophosphamide [66] and potentially thalidomide [67, 68] do not cause congenital anomalies. This is unlike in pregnant women where there is greater potential for teratogenic effects from some antirheumatic drugs. It is therefore suggested that male patients with active disease remain on their antirheumatic medications, the exception being in the use of cyclophosphamide and thalidomide, while attempting conception. In such patients, the risk of stopping medications may have a substantial effect.

### Contraception and preservation of fertility

Advice from the WHO and CDC guidelines [69] is that barrier methods of male contraception can be a safe method and that using condoms should be reinforced. This is especially relevant to those males taking biological and other immunosuppressive medications. However, barrier methods, especially in teenagers, have high failure rates when this is the single method of contraception [70]. Therefore, in order to avoid pregnancy, it is mandatory to suggest more reliable contraception technique in conjunction with barrier methods.

Semen cryopreservation, a well-established effective approach, is one of a number of strategies explored to preserve fertility in men receiving cytotoxic medications. On the other hand, the use of gonadotropin-releasing hormone (GnRH) agonists to suppress the pituitary-gonadal axis has not shown benefit in studies carried out on humans [71].

### Approach to management

#### Multidisciplinary approach to sexual dysfunction in men

Due to the multiplicity and complexity of forms of sexuality expression among men living with ARDs, the approach of this cohort with sexual dysfunction involves broad aspects and hard-to-approach themes, whose handling requires the formation of bonds and an environment enabling the understanding of the physical complaints aspects and beyond, in particular, the emotional and social factors [72, 73].

The rheumatology-led pregnancy clinic represents the main gate for the appropriate patients’ care delivered by a multidisciplinary team. Such approach permits the set-up and initiation of actions at different levels of complexity in health care. In this perspective, the psychologist would be the most appropriate speciality to provide management for the emotional problems, related to the illness process, and the implications of these issues on the affective and sexual relationship with the patient [73, 74]. Interventions to control pain and increase mobility and muscle strength, providing improved physical capacity for the patient, are held by the physical therapist, and this process is monitored by a physical education professional [75]. This will facilitate the improvement of objective symptoms linked to the disease, such as fatigue, pain and joint movement restrictions. Guidelines on the organization of the routine and protection of joints during activities of daily living, as well as the indication of assisted technology to modify objects and environments, are demands met by occupational therapists. Simple baseline measurements and investigations can be initiated in the rheumatology clinic and give guidance to the next step of management [76].

#### Self-management approaches

Different ways have been described discussing how to handle sexual function and sexual relationship problems in ARDs patients. Examples are, finding the best possible conditions to facilitate sexual arousal, best, least painful, position for making love or how to handle hand pain. These examples are in line with the strategies for self-management which Helland et al. [73] have described. These suggestions included being creative during the sexual act, adapting positions and movements; using alternative locations; using painkillers or pillows; ignoring restrictions; initiating less strenuous sexual activity; postponing sexual activity until flares have passed and having sex despite a lack of desire. Treating health care professionals/rheumatology nurses, should leave the door open for the patient to confer any worries or future plans, he might have. With this in the background, it has been suggested to include self-management strategies in the standard clinical care of ARDs patients. In a study carried out by Stoffer et al. [77] as well as in earlier research (National Rheumatoid Arthritis Society) [78], the cohort of patients included in these works, raised their concerns that the their treating healthcare professionals did not include any form of evaluation or interventions in relation to their sex life. According to Matheson et al. [79], when managing inflammatory arthritic conditions, it is vital to involve the partner in any discussion, to be able to provide comprehensive supportive care for patients as well as their families. To achieve this, it is important to pay attention to any sexual difficulties the patient might have or develop during the clinical follow-up visits. Also, whenever appropriate, to offer sexual counseling to the patient and his partner [80].

#### How the rheumatologists can guide their male patients

Though significant percentage of men living with ARD or receiving medical management for their disease, have reported negative impact of their disease or medication on their sexual health, yet, in majority the cases, the patients have not raised this problem for discussion with their treating health professional [25, 81]. In fact, in standard clinical practice, most of the time, sexual problems are ignored. Furthermore, patients with inflammatory arthritis in general and men in particular, vary in their preference of health professional with whom to discuss these issues [2], suggesting all health care professionals whether rheumatologists or rheumatology nurses involved in men care should attain better knowledge and understanding of how inflammatory arthritis can impact on both family planning and sexual function.

Sexual dysfunction is closely related to disease activity, its medical therapy and associated comorbidities. Table 2 depicts an approach to assessment of the male sexual health problems and its associated comorbidities which can be implemented in standard practice. To facilitate the process of early detection, it is advisable that a standard core theme should be implemented, bearing in mind how such a sensitive and private problem can be handled in standard clinical practice. In a study using a multidimensional PROMs questionnaire [2], which included questions regarding sexual dysfunction, the individual patient had the opportunity to highlight these problems. This helped both the patient and healthcare physician to bridge the gap and open this sensitive topic.

**Table 2:** Approach to assessment of the male sexual health problems and its associated comorbidities which can be implemented in standard practice

Associated condition	Assessment		
	History and clinical examination	Measurement	Lab tests
Cardiovascular disease	√	Blood pressure	
Hypertension	√	Blood pressure	
Diabetes mellitus			HbA_1_c
Endocrine disorders (e.g., hypogonadism, hyperprolactinemia, thyroid disorders)	√		Morning testosterone, prolactin, TSH
Genital pain	√		
Hyperlipidemia		ECG	Lipid profile
Metabolic syndrome	√		Blood pressure; HbA_1_c, high-density lipoprotein, and triglyceride levels; waist circumference
Neurologic conditions (e.g., multiple sclerosis, Parkinson disease, spinal cord injury, stroke)	√		
Obesity	√	Body mass index, waist circumference	
Prostate cancer treatment (e.g., surgery, radiation, hormone therapy)	History		PSA, US
Psychological conditions (e.g., anxiety, depression, guilt, history of sexual abuse, marital or relationship problems, stress)	History		
Sedentary lifestyle	History		
Smoking	History		
Trauma	History		
Venous leakage	√	Urology consultation for venous flow testing	

Panush et al. [82] described a strategy to approach and offer guidance on sexual function, called as PLIS-SIT (permission, limited information, specific strategies and intensive therapy). Permission consists in questioning the patient about his/her sexual dysfunction, taking the liberty and showing openness to dialog. The second step is to search and provide information about sexual dysfunction. The third phase is to develop specific strategies for each problem. In case of arthritic men who develop impotence, usually of psychogenic origin, phosphodiesterase inhibitors may be used, with “A” level of evidence in cases of organic, psychogenic and pharmacological erectile dysfunction [83]. The fourth step involves referring the patient to the sex therapist, in case of failure of other strategies.

##### Algorithm of management

**Fig. 2:**
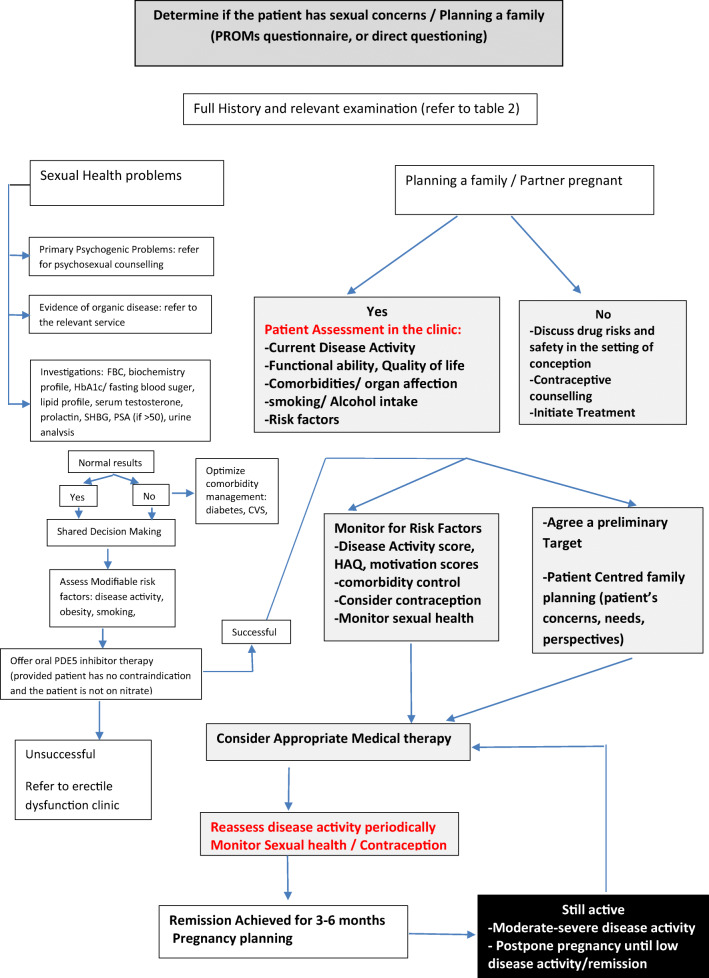
Flowchart describing a proposed set-up of the pregnancy clinic and how to evaluate men living with autoimmune rheumatic diseases and presenting with sexual problems/concerns or planning for a family

In order to conduct an accurate evaluation of a male patient it is essential to carry out a full medical assessment including: medical and surgical history, use of medications and other substances, sexual history, and evaluation for both psychological and relationship health. Physical examination should include assessment of body mass index and waist circumference to assess abdominal obesity and male secondary sex characteristics as well as taking the blood pressure. Figure 2 shows a flow chart describing a proposed set-up of the pregnancy clinic and how to evaluate men living with autoimmune rheumatic diseases and presenting with sexual problems/concerns or planning for a family

There is some controversy around the use of testosterone measurement in men with erectile dysfunction (ED) and that a diagnosis of hypogonadism must be based on more than just an abnormal laboratory test result. In cases where the morning total testosterone level is repeatedly normal while hypogonadism is clinically suspected, measuring bioavailable or free testosterone would be the next step [84]. To be able to differentiate between primary and secondary causes of testicular hypogonadism, it is advisable to measure the levels of follicle-stimulating hormone, luteinizing hormone, sex hormone–binding globulin, estradiol, and prolactin in the patient’s blood [85].

Strategies for contraception and preservation of fertility need to be considered wherever appropriate for the group of male patients at high risk. For those being treated with cytotoxic medications, measures for preservation of fertility include, primarily, the well-established effective approach of semen cryopreservation. While the use of gonadotropin-releasing hormone (GnRH) agonists, to suppress the pituitary-gonadal axis, has not shown benefit in human studies [86], assisted reproductive technologies (ART) such as in vitro fertilization with intracytoplasmic sperm injection (ICSI) resulted in successful pregnancies, particularly for those cases with much lower concentrations of sperms than normal. Evaluation should be carried out for men with azoospermia to assess whether there are spermatozoa which could be taken for ICSI directly from the testis. ART approach can be considered for many medications, while the patient is taking or not taking the treatment, the exceptions to this would be thalidomide and cyclophosphamide.

Men may continue sulfasalazine (SSZ) therapy while pursuing pregnancy; however, in those men where several attempts at pregnancy have been unsuccessful, a semen analysis would need to be undertaken. Subsequently, if the results of the semen analysis were abnormal, stopping medication for three to four months may be advised to allow time for the recovery of spermatogenesis [42, 87].

To avoid the risk of teratogenicity, it is recommended that Cyclophosphamide should be stopped for at least three months before attempting conception as this would allow for a complete cycle of spermatogenesis to take place. It is also recommended that Thalidomide needs to be discontinued for 4 weeks before attempting conception [41].

## Conclusion

Although there is a high prevalence of sexual problems, which have a huge impact on the lives of men living with rheumatic diseases, this has had very little attention. Additionally, preceding research has not considered the multifaceted aspects and complex nature of sexual problems in the context of ARDs. Treating rheumatologists/rheumatology nurses must take into account family planning for both men and women attending their ARD clinics. Rheumatology-led pregnancy clinic is a good option that should be adopted in every rheumatology practice. In-depth counseling and support are required to agree a treatment strategy tailored to the patient’s plans and expectations, guide the patients with their appropriate therapies, best approach to contraception, and answer their queries. This emphasizes the value of reporting pregnancy outcomes to help and guide patients in the future.
